# ASCIZ/ATMIN is dispensable for ATM signaling in response to replication stress

**DOI:** 10.1016/j.dnarep.2017.06.022

**Published:** 2017-09

**Authors:** Rui Liu, Ashleigh King, Nicolas C. Hoch, Catherine Chang, Gemma L. Kelly, Andrew J. Deans, Jörg Heierhorst

**Affiliations:** a St. Vincent’s Institute of Medical Research, 9 Princes Street, Fitzroy, Victoria 3065, Australia; b Department of Medicine (St. Vincent’s Health), The University of Melbourne, 41 Victoria Parade, Fitzroy, Victoria 3065, Australia; c Walter and Eliza Hall Institute of Medical Research, Parkville, Victoria 3052, Australia; d Department of Medical Biology, The University of Melbourne, Parkville, Victoria 3052, Australia

**Keywords:** MEFs, murine embryonic fibroblasts, Aphidicolin, ASCIZ, ATM, ATMIN, DYNLL1, 53BP1

## Abstract

The ATM kinase plays critical roles in the response to DNA double-strand breaks, and can also be activated by prolonged DNA replication blocks. It has recently been proposed that replication stress-dependent ATM activation is mediated by ASCIZ (also known as ATMIN, ZNF822), an essential developmental transcription factor. In contrast, we show here that ATM activation, and phosphorylation of its substrates KAP1, p53 and H2AX in response to the replication blocking agent aphidicolin was unaffected in both immortalized and primary ASCIZ/ATMIN-deficient murine embryonic fibroblasts compared to control cells. Similar results were also obtained in human ASCIZ/ATMIN-deleted lymphoma cells. The results demonstrate that ASCIZ/ATMIN is dispensable for ATM activation, and contradict the previously reported dependence of ATM on ASCIZ/ATMIN.

## Highlights

• ASCIZ/ATMIN is not required for ATM activation by replication stress in MEFs.
• ATM activation is normal in human ASCIZ/ATMIN KO cells.
• ASCIZ/ATMIN is dispensable for aphidicolin-induced 53BP1 focus formation.

## 1 Introduction

ATM/ATR-like kinases play central roles in the cellular response to DNA damage [1]. ATM primarily functions in the response to DNA double-strand breaks [1], but can also respond to oxidative stress [2], hypoxia [3], chromatin perturbations [4] and prolonged replication blocks [5]. Whereas canonical activation of ATM by DNA double-strand breaks depends on its association with the Mre11-Rad50-Nbs1 (MRN) complex [6], it has been proposed that ATM activation by chromatin perturbations and replication stress is mediated by its association with an alternative activator, ASCIZ/ATMIN [7], [8], [9], [10].

ASCIZ (ATM substrate Chk2-interacting Zn^2+^-finger protein) was originally identified in our laboratory as a protein involved in the response to DNA base damage [11]. ASCIZ forms nuclear foci in cells treated with MMS or H_2_O_2_ [11], [12], and its loss leads to mildly increased cell death in response to these agents in human, mouse and chicken cells [11], [13], [14], [15], [16]. *Drosophila* ASCIZ also relocalizes to laser-induced oxidative damage-mimicking lesions [17], and *dAsciz* heterozygous flies are hyper-sensitive to oxidative stress [18]. While these data indicate that ASCIZ plays a highly conserved role in the response to DNA base damage, the molecular mechanisms involved in this function remain unclear. However, it has recently become clear that ASCIZ has major DNA damage-independent functions as an essential Zinc-finger transcription factor. ASCIZ activates the expression of the multi-functional dynein light chain, DYNLL1 (also known as LC8) [19], and thereby regulates crucial developmental processes, including the earliest stages of embryonic lung formation [13], [20], B cell development [21] and B cell lymphomagenesis [22], and the production of myeloid cells [23].

Following its identification as a DNA damage response protein, ASCIZ was renamed ATMIN (ATM interactor) based on a report that ASCIZ/ATMIN and ATM were required for each other’s stability [7] (akin to the reciprocal stabilization of the related ATR kinase and its regulatory subunit ATRIP [24]). However, this finding could not be reproduced in several subsequent studies [8], [13], including by the same authors [10], [25], [26]. Nonetheless, it was reported that ASCIZ/ATMIN was specifically required for ATM activation in response to chromatin perturbations [7], [25], and DNA replication blocks [8], [9], [10].

Here we have re-evaluated the requirement of ASCIZ/ATMIN for ATM activation by replication blocks using similar but independently derived *Asciz/Atmin*-deficient cells. Using several independent lines of experimentation, we show that ATM activation by the replication blocking agent aphidicolin was normal in the complete absence of ASCIZ/ATMIN protein. These results demonstrate that ASCIZ/ATMIN is not required for ATM activation, and indicate that unrelated genetic changes in the ASCIZ/ATMIN-deficient cell line used in the earlier reports may have indirectly affected the non-canonical ATM activation pathway.

## 2 Materials and methods

### 2.1 Immortalized murine embryonic fibroblasts (MEFs)

MEFs were prepared from E12.5-E14.5 embryos collected over a two-month period in 2009, and were spontaneously immortalized using a 3T3 protocol as described [13]. The following MEF cell lines were used for the experiments reported here (numbers designate the originating embryo): wildtype: MJI-65, MJI-91, MJI-105; *Asciz^−/−^*: MJI-1, MJI-84, MJI-86.

### 2.2 Primary MEFs

Animal experiments for the isolation of primary MEFs were approved by the St. Vincent’s Hospital Melbourne Animal Ethics Committee. Primary MEFs were freshly prepared from four E14.5 embryos (2 wildtype, 2 *Asciz^−/−^*) obtained from the same litter for Western blot analyses, and six additional embryos (2 wildtype, 1 heterozygote, and 3 *Asciz^−/−^*) from 3 different litters for immunofluorescence microscopy experiments. Embryos were dissected in ice-cold PBS, and after removal of heads and internal organs, were minced with a scalpel, trypsinized and seeded overnight in Dulbecco’s Modified Eagles Medium containing 10% fetal calf serum (DMEM-FCS) in 10-cm tissue culture dishes. Non-adherent cells were removed by washing with PBS, and cells were cultured without trypsinization for a further two days. Cells were then washed, trypsinized, Coulter-counted, and used for experiments.

### 2.3 Inducible CRISPR/Cas9-mediated deletion of ASCIZ in human cells

The human Burkitt lymphoma cell line BL30 was sequentially transduced with lentiviral expression vectors for constitutive expression of Cas9 and inducible expression of guide RNAs as described [27]. The guide RNA sequence for human *ASCIZ* was 5′- AGTGCTGTTCTACTGGCGTA-3′. As a non-targeting control, an inducible guide RNA against mouse *Bim* exon 3 (5′- GCACAGGAGCTGCGGCGGAT −3′) was used, which does not recognize the human *BIM* gene.

### 2.4 Cell culture and DNA damage treatments

For immunofluorescence experiments 1.5 × 10^5^ MEFs per well were seeded in 6-well plates on sterilized coverslips, and grown for 2 days in 3 ml DMEM-FCS. For western blot analyses, 1 × 10^6^ MEFs were seeded per 10-cm dish in 10 ml DMEM-FCS. After a PBS wash, fresh medium was added for 24 h. Aphidicolin (Sigma, A0781) was added at 1–3 μM as indicated in the figure legends. For comparison, some cultures were irradiated with 2 Gy after 23 h, followed by 30-min recovery. Human BL30 cells were cultured in RPMI-1640, 10% FCS, 1 mM glutamine, 1 mM sodium pyruvate and 50 μM thioglycerol in 10-ml flasks. Cells were treated with 1 μg/ml doxycycline hyclate (Sigma-Aldrich) for 60 h, and then diluted three-fold into fresh medium containing 1 μg/ml doxycycline and 3 μM aphidicolin for a further 36 h. BL30 control cultures lacking doxycycline and/or aphidicolin treatments were processed in parallel. All cells were incubated at 37 °C with ambient O_2_ and 5% CO_2_.

### 2.5 Western blot analyses

MEFs were scraped into cold modified RIPA buffer (150 mM NaCl, 20 mM Tris pH7.4, 1 mM EDTA, 1 mM EGTA, 10 mM NaF, 1% Triton X-100, 1% sodium deoxycholate, 0.1% SDS, 1 mM PMSF, 1 x protein inhibitor cocktail (Sigma)), lysed on ice, sonicated, and centrifuged for 10 min at 4 °C. For subcellular fractionation analyses, cells were trypsinised and processed using a Cell Fractionation kit (Abcam, ab109719) following the included protocol. Human BL30 cells were collected by centrifugation at 375 x *g*, washed in ice-cold PBST, and lysed as above. 10–15 μg protein per lane were electrophoresed and transferred to PVDF membranes for detection using ECL (GE Healthcare) or Super-ECL reagents (Pierce) and X-ray film (Fuji). Films were scanned in trans-illumination mode using a dual lens Epson V700 photoscanner, and band intensities were quantified using ImageJ software (NIH, Bethesda). The following antibodies were used for western blots: Actin (EMD Millipore/Merck, MAB1501), ATM (Abcam, 5C2, ab2618), DYNLL1 (Abcam, ab51603), FANCD2 (Abcam, ab178705), 53BP1 (Novus, NB100-304, lot A3), γH2AX (EMD Millipore/Merck, 05-636), H2AX (Abcam, ab20669), KAP1 (Proteintech, 15202-1-AP), M601 (Abcam, ab110411), p53 (Cell Signaling Technology, 1C12; for mouse samples), p53 (Santa Cruz Biotechnology, DO-1, sc-126; for human samples), phospho-CRMP2 (Ser522; ECM Biosciences, CP2191), phospho-ATM (Ser1981; Cell Signaling Technology, 10H11.E12), phospho-KAP1 (Ser824; Bethyl, A300-767A); phospho-p53 (Ser-15; Cell Signaling Technology, 9284); α-tubulin (Sigma-Aldrich, T9026).

### 2.6 Immunofluorescence

Cells were fixed in 2% para-formaldehyde in PBS for 10 min; rinsed in PBS; incubated for 1 min in ice-cold methanol; rinsed in PBS; blocked overnight in PBS containing 10% horse serum; incubated with 53BP1 antibody (1/400 dilution; Novus, NB100-304, lot B1) for 4 h; washed 3 times in PBS-0.1% Tween-20; incubated with 1/500 diluted goat anti-rabbit Ig-Alexa-594 (Thermo Fisher) for 1 h; washed 3 times in PBS-0.1% Tween-20; and coverslips were mounted using 7 μl Antifade (DAKO) containing 1 μg/ml DAPI. Micrographs were taken using a Zeiss Axiovert-25 microscope with a Zeiss AxioCam MRc camera using a 40x objective. Images were analysed using AxioVision software and at least 200 cells per slide were scored.

## 3 Results

### 3.1 ASCIZ is not required for ATM activation in response to replication stress in immortalized fibroblasts

**Fig. 1:**
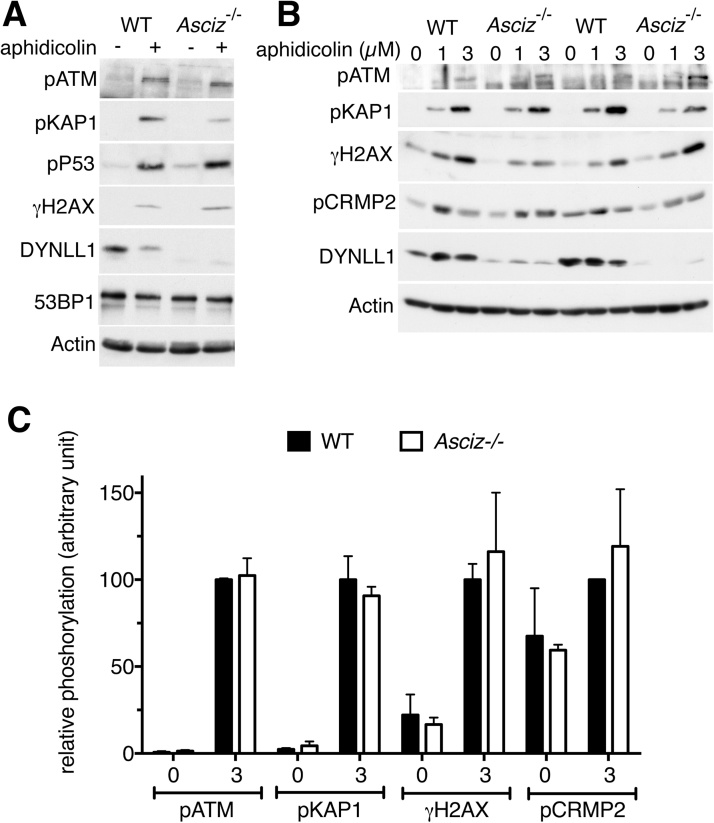
Aphidicolin-induced ATM signaling in immortalized MEFs. (A) Cells (WT, MJI-53; KO, MJI-1) were treated for 24 h using 0 or 3 μM aphidicolin. (B) Cells (lane 1–3, WT, MJI-91; lanes 4–6, KO, MJI-186; lanes 7–9, WT, MJI-105; lanes 1–12, KO, MJI-84) were treated for 24 h using 0, 1 or 3 μM aphidicolin. Actin and 53BP1 serve as loading controls. DYNLL1 is used as a surrogate marker for loss of ASCIZ. (C) Quantification of western blot band intensities. An arbitrary unit of 100 represents the average band intensity for the respective phospho-protein in the 3 μM aphidicolin-treated wildtype samples on each membrane. Graphs indicate the mean ± standard error, n = 3. Additional loading controls for total KAP1, p53 and H2AX are shown in Supplementary Fig. S1A and B.

It has recently been reported that ATM activation (measured by Ser1981 phosphorylation) in response to the replication blocking agent aphidicolin, and phosphorylation of its substrates H2AX, KAP1 and p53, was dramatically impaired in an immortalized murine embryonic fibroblast (MEF) cell line from *Asciz/Atmin* knockout mice compared to matched control MEFs [8], [9]. We were intrigued by the severity of this defect, and attempted to replicate these findings using our own *Asciz/Atmin* knockout MEFs under similar experimental conditions. Surprisingly, we found that aphidicolin-dependent phosphorylation of ATM (pSer1981), KAP1 (pSer824), p53 (pSer15) and H2AX (γH2AX, pSer140 in mice) was not substantially affected in an *Asciz*-null MEF cell line compared to a control MEF cell line, which had been immortalized around the same time from a wildtype embryo obtained from a similar *Asciz^+/−^* X *Asciz^+/−^* mating pair (Fig. 1A). Based on this striking discrepancy to the previous reports, we repeated this experiment using additional, independently derived MEF cell lines from two more *Asciz^−/−^* and two more wildtype embryos, with similar results (Fig. 1B). Altogether, these results consistently showed that ATM activation and phosphorylation of its substrates was not impaired in the three independent *Asciz*-null cell lines compared to the three different control cell lines (Fig. 1C).

### 3.2 ASCIZ is not required for replication stress-dependent ATM activation in primary MEFs

Freshly isolated MEFs enter senescence after ∼20–30 generations in culture, and their spontaneous immortalization depends on the acquisition of random mutations (usually in the ATM-related p53 pathway), which overcome the senescence-associated cell cycle arrest and allow cells to resume proliferation [28], [29]. As such, one possible explanation for the difference between our, and the previously reported results was that coincidental, random genetic changes were selected during the immortalization process. Such changes then may have either indirectly interfered with the normal ATM activation process (in the case of the MEF cell line sourced from the Behrens laboratory), or may have indirectly restored an otherwise defective ATM activation mechanism (in the cases of all three of our independent *Asciz*-null MEF cell lines). Thus, to exclude any possibility of selection artefacts, we used freshly isolated and minimally cultured primary MEFs from two additional *Asciz^−/−^* embryos, and two wildtype littermate controls, to repeat these experiments.

**Fig. 2:**
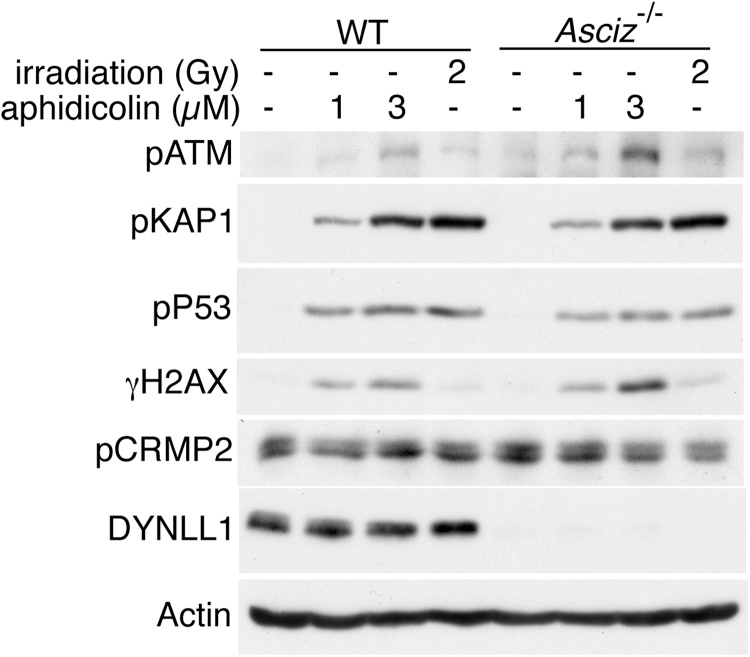
Aphidicolin-induced ATM signaling in primary MEFs. Freshly prepared primary MEFs from a wildtype and an *Asciz^−/−^* embryo were treated for 24 h with 0, 1 or 3 μM aphidicolin. For comparison, cultures from the same embryos were also irradiated with 2-Gy and allowed to recover for 30 min. Similar results for another independent pair of wildtype and *Asciz^−/−^* primary MEFs are shown in Supplementary Fig. S1C, and additional loading controls for total KAP1, p53 and H2AX are shown in Supplementary Fig. S1D.

In these primary MEFs, we observed robust phosphorylation of ATM and its substrates after treatment with aphidicolin, which was comparable to or even more pronounced than the canonical activation of ATM in response to 2-Gy γ-irradiation of the same cultures (Fig. 2 and Supp. Fig. S1C). However, similar to our immortalized MEFs, there was no reduction in the replication stress-induced activation of ATM in the primary *Asciz*-null MEFs compared to their wildtype littermate controls (Fig. 2 and Supp. Fig. S1). Thus, these experiments conclusively demonstrate that ASCIZ/ATMIN is not required for ATM activation in response to replication stress.

Fig. S1

### 3.3 Replication stress promotes ATM-independent CRMP2 phosphorylation only in immortalized but not in primary MEFs, and this does not depend on ASCIZ

In their proteomics analysis of the aphidicolin response, Mazouzi et al. [9] reported that ASCIZ/ATMIN was required for the ATM-independent phosphorylation of several proteins, most notably Ser522 phosphorylation of the microtubule-associated protein CRMP2 by CDK5 or GSK3. CRMP2 has been shown to interact with the cytoplasmic and intra-flagellar dynein motor complexes [30], [31], [32]. Given that ASCIZ/ATMIN regulates essential developmental processes as a transcription factor for the dynein subunit DYNLL1 [19], [20], this raised the exciting possibility that altered CRMP2 phosphorylation may be a functionally important downstream effector of the ASCIZ-DYNLL1 axis. To explore this, we monitored CRMP2 phosphorylation in our MEFs. Similar to Mazouzi et al. [9], we observed increased CRMP2 (pSer522) phosphorylation in immortalized MEFs after 24 h of aphidicolin treatment (Fig. 1B, C). However, similar to our findings for the ATM pathway, aphidicolin-induced CRMP2 phosphorylation was not reduced in the three *Asciz*-null MEF cell lines compared to their matched controls (Fig. 1B, C). Moreover, in primary MEFs, CRMP2 was already highly phosphorylated under basal conditions, and its phosphorylation was not further increased in response to aphidicolin treatment, in both *Asciz*-null cells and littermate control cells (Fig. 2 and Supp. Fig. S1). Altogether, these results demonstrate that ASCIZ is also dispensable for the proposed ATM-independent component of the replication stress response.

### 3.4 ASCIZ/ATMIN is not required for 53BP1 focus formation

**Fig. 3:**
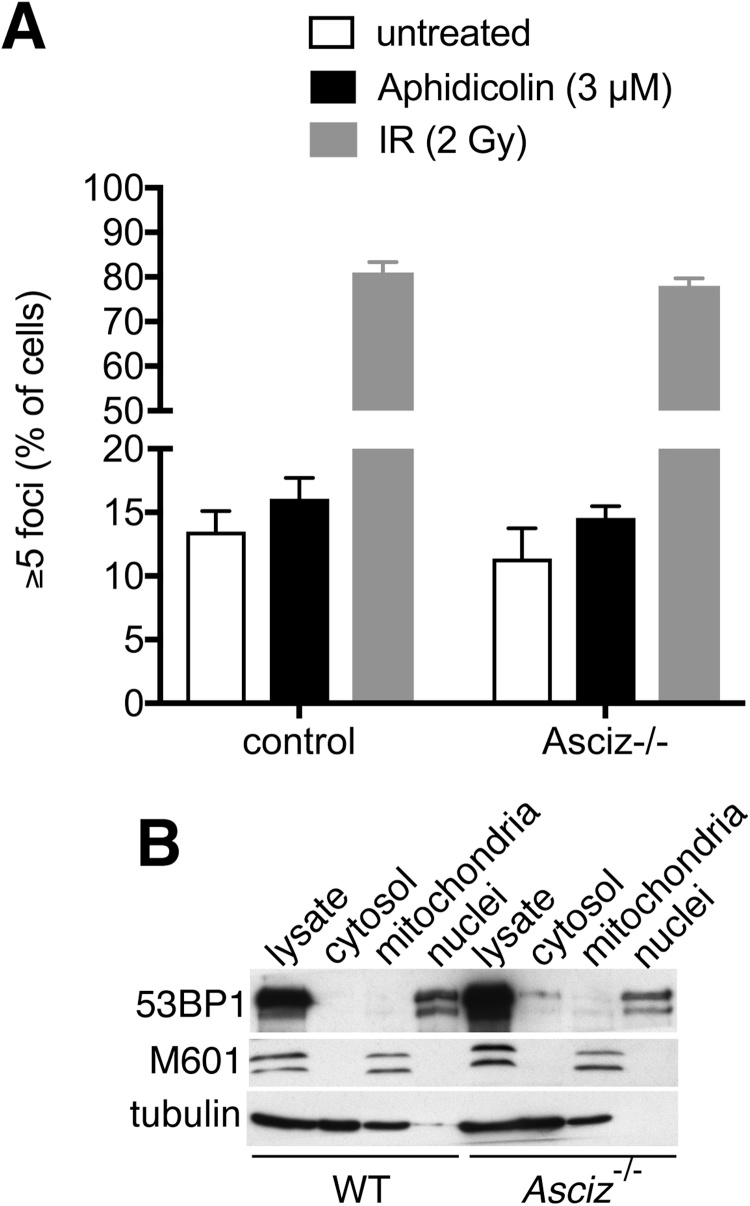
53BP1 focus formation. (A) Bars indicate the mean ± standard error of 53BP1 focus-forming cells in three independent control and three independent *Asciz^−/−^* primary MEF cultures under basal conditions (open bars), in response to 24-h aphidicolin treatment (black bars) and following 30 min recovery after ionizing radiation (grey bars). More detailed quantifications of foci/cell are shown in Supp. Fig. S2. (B) Subcelllular distribution of 53BP1 in cell fractionation assays. M601 is used as a mitochondrial marker, and tubulin as a cytosolic marker.

Unresolved replicative DNA damage can lead to ATM-dependent 53BP1 focus formation in the subsequent G1 phase [5]. This has been reported to be reduced in *Asciz/Atmin*-null MEFs [8], [10]. We therefore also assessed 53BP1 focus formation in freshly isolated primary MEFs from two control and two *Asciz^−/−^* embryos (Fig. 3A). In the aphidicolin-treated control cultures, we noticed a small increase in cells with five or more 53BP1 foci compared to untreated samples (from 13.5% to 16.1% of cells; note that we did not observe an increase in focus-forming cells if a lower threshold of foci/cell was used (Supp. Fig. S2)). In aphidicolin-treated *Asciz^−/−^* cultures, there was a similar increase in the percentage of 53BP1-focus forming cells (from 11.4% under basal conditions to 14.6% of cells after aphidicolin treatment). For comparison, ∼80% of control and *Asciz^−/−^* cells formed five or more 53BP1 foci in response to ionizing radiation (Fig. 3A). Finally, consistent with previous reports that the ASCIZ target DYNLL1 is a major 53BP1-binding protein [33] and may be involved in its nuclear import [34], we noticed a modest redistribution of 53BP1 towards the cytoplasm in primary *Asciz^−/−^* MEFs compared to littermate control cells in subcellular fractionation assays (Fig. 3B). Nevertheless, although the basal percentage of 53BP1 focus-forming cells was slightly lower in *Asciz^−/−^* cells, collectively, our data indicate that ASCIZ/ATMIN is not required for the regulation of 53BP1 focus formation in response to aphidicolin-induced replication stress.

Fig. S2

### 3.5 ASCIZ/ATMIN is not required for aphidicolin-induced ATM activation in human cells

**Fig. 4:**
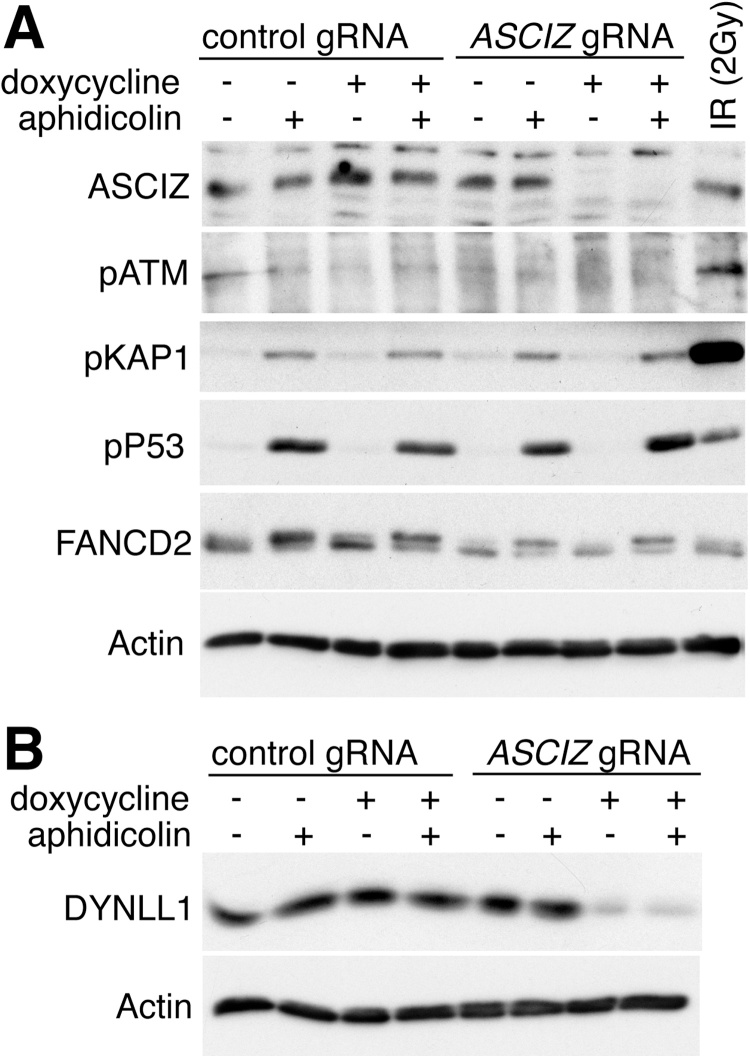
Aphidicolin-induced ATM signaling in human ASCIZ/ATMIN knockout cells. (A, B) BL30 cells were cultured as indicated in the presence or absence of doxycycline to induce a non-targeting control guide RNA or the guide RNA against *ASCIZ*, and aphidicolin to elicit prolonged DNA replication stress as indicated. For comparison, control guide RNA transduced cells were also irradiated for canonical ATM activation without doxycycline or aphidicolin treatment in panel A. Panel B shows a blot of the same samples except the irradiated control. Additional loading controls for total ATM, KAP1 and p53 are shown in Supplementary Fig. S1E.

To determine if these findings extend beyond mouse cells, we utilized CRISPR/Cas9 technology to delete *ASCIZ/ATMIN* in human cells. To prevent selection of random genetic alterations in *ASCIZ/ATMIN* knockout cell lines, we adopted a doxycycline-inducible CRISPR/Cas9 approach that has recently been optimized for human Burkitt lymphoma cell lines [27]. Upon induction of the *ASCIZ/ATMIN* guide RNA for four days, ASCIZ protein was undetectable in the BL30 cell line (Fig. 4A), and DYNLL1 protein levels as a surrogate marker for ASCIZ depletion were also reduced by ∼10-fold compared to control cells (Fig. 4B). In BL30 cells, ATM autophosphorylation in response to aphidicolin treatment was too weak to be detectable by Western blot, relative to the canonical ATM activation by ionizing radiation (Fig. 4A). However, phosphorylation of the more sensitive ATM-specific KAP1 serine-824 site, and p53 serine-15 as a general replication stress marker, in response to prolonged aphidicolin treatment was not reduced in the *ASCIZ/ATMIN* knockout cells compared to the same cells cultured in parallel without doxycycline-induced guide RNA expression, and control cells expressing a non-targeting guide RNA (Fig. 4A). In addition, in contrast to the findings of Schmidt et al. [8], the mono-ubiquitination-dependent electrophoretic mobility shift of FANCD2 in response to aphidicolin treatment was also not increased in the *ASCIZ/ATMIN*-deleted cells (Fig. 4A). Thus, these data demonstrate that loss of ASCIZ/ATMIN does not affect non-canonical ATM activation in response to prolonged replication stress in human b cell lymphoma cells.

## 4 Discussion

In contrast to other recent reports [8], [9], the data presented here demonstrate that ASCIZ/ATMIN is not required for ATM signaling in response to the replication blocking agent aphidicolin in murine or human cells. These results are consistent with our previous report, using primary MEFs from different embryos than those used here, that ASCIZ/ATMIN was also dispensable for ATM activation in response to a different replication blocking agent, hydroxyurea (Supplemental Fig. S3 in [13]). These contradictory findings for the replication stress response extend our findings that, in contrast to [7] and [25], ASCIZ/ATMIN was also not required for non-canonical ATM activation by chloroquine-induced or hypotonic chromatin perturbations in MEFs (primary and immortalized) and B lymphocytes from our *Asciz*-null mice [13], [22], as well as in siRNA-treated human ASCIZ knock-down cell lines [13]. Furthermore, our results are also consistent with a recent report that ASCIZ/ATMIN was not required for the non-canonical ATM activation by another stimulus, hypoxia [15].

The *Asciz/Atmin* null alleles from the different laboratories are almost identical (removal of an exon encoding the C-terminal 601 of 818 amino acid residues [7], [13]). The two mouse strains are on different genetic backgrounds (inbred C57BL/6 for our mice vs mixed BALB/c*C57BL/6 for the mice from the Behrens laboratory), but it is highly unlikely that this difference alone could explain the extreme differences with regard to ATM activation. As discussed above, MEFs incur incidental genetic changes during the immortalization process, most frequently in the ATM-related p53 pathway. Our findings are consistent for several independent immortalized MEF cell lines as well as for several independent minimally cultured primary cells preparations. In contrast, the reports supporting a role for ASCIZ/ATMIN in ATM activation by chromatin perturbations or replication blocks seem to be based on immortalized MEFs [7], [8], [9], and as far as we can tell from their description in the literature [7], [9] may have involved a single *Asciz/Atmin*-null cell line. The simplest explanation for the observed differences is, therefore, that the cell line used in the other studies may have contained an unrelated mutation, which may then have interfered with the non-canonical activation of ATM in an ASCIZ/ATMIN-independent manner. In this context, it is noteworthy that the *Asciz/Atmin*-null MEFs used by Mazouzi et al. have a very different morphology and cytoskeletal architecture compared to their wildtype control MEFs (see Fig. 6B in [9]).

While our data demonstrate that non-canonical ATM activation was normal in human *ASCIZ/ATMIN*-knockout lymphoma cells (Fig. 4), we acknowledge that reduced ATM activation in response to aphidicolin treatment has recently been reported in human *ASCIZ/ATMIN* siRNA knockdown cell lines [10], [15]. However, acute siRNA knockdown of *ASCIZ/ATMIN* in adherent human cell lines can lead to a marked reduction of cell proliferation (measured by BrdU incorporation for S phase cells by us [11] and phospho-histone H3 staining for mitotic cells by [7]), which consequentially would reduce the overall level of replication stress in a given cell population. It should also be noted that in one of these studies, the aphidicolin-induced phosphorylation of ATM, KAP1 and p53 was only very modestly reduced in ATMIN/ASCIZ-depleted cells compared to the near-complete inactivation of the ATM pathway after depletion of RAD18 and WRNIP1, which were proposed to regulate replication stress-induced ATM activation in a common pathway, or in complex with ASCIZ/ATMIN [10].

In summary, based on consistent results from several independent laboratories, it now appears to be universally accepted that ASCIZ/ATMIN plays a key role as an essential transcription factor by regulating the expression of DYNLL1 [15], [17], [19], [20], [21], [22], [23]. In contrast, its role in the ATM activation process remains controversial [35]. We feel it is important to bring our negative results to the attention of the scientific community, in the hope that this may facilitate critically informed experimental analyses of non-canonical ATM activation pathways.

## Author contributions

RL, AK, NCH and JH designed, performed, analysed and discussed experiments; CC and GLK provided reagents; AJD discussed experiments; JH wrote and NCH and AJD edited the manuscript.

## Conflicts of interest

The authors declare that there are no conflicts of interest.
