# Supplementary material for: ASCIZ/ATMIN is dispensable for ATM signaling in response to replication stress
Source: DNA Repair (Amst). 2017 Sep;57:29–34. doi: 10.1016/j.dnarep.2017.06.022 (PMC5576915; doi:10.1016/j.dnarep.2017.06.022)
Supplement: Fig. S1 [file mmc1.pdf]

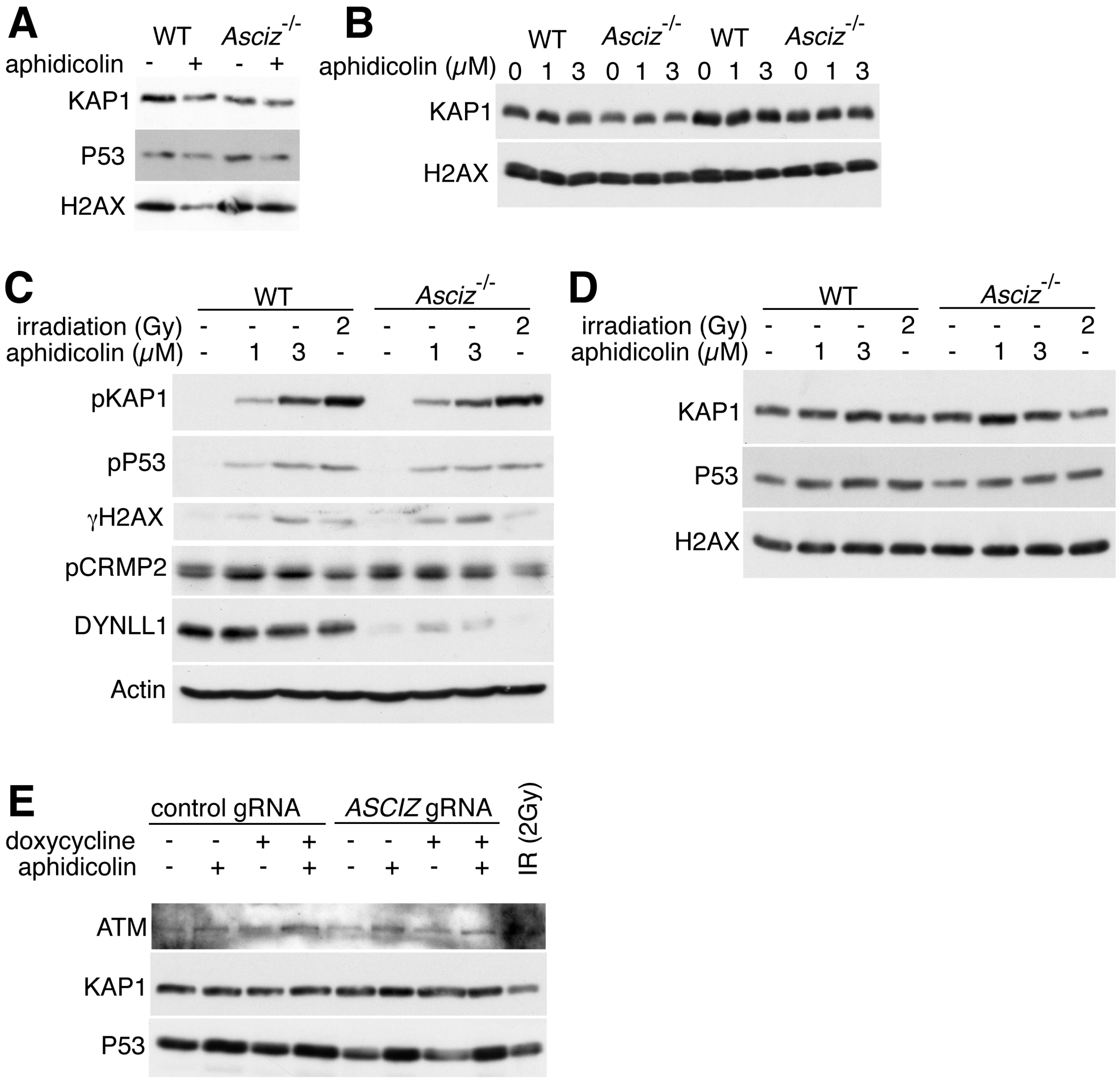

Supplementary Figure S1. Additional Western blots. (A, B) Loading controls for total KAP1, p53 and H2AX for Fig. 1A, B. Identically loaded samples were analysed on new blots. (C) Aphidicolin-induced ATM signaling in primary MEFs. Freshly prepared primary MEFs from a wildtype and an *Asciz*$^{-/-}$ embryo were treated for 24 hours with 0, 1 or 3 μM aphidicolin, or irradiated with 2-Gy. These MEFs were derived from independent embryos in parallel with the cells shown in Fig. 2. (D) Loading controls for total KAP1, p53 and H2AX for Fig. 2. Identically loaded samples were analysed on new blots. (E) Loading controls for total ATM, KAP1 and p53 for Fig. 4.
