# Supplementary material for: ASCIZ/ATMIN is dispensable for ATM signaling in response to replication stress
Source: DNA Repair (Amst). 2017 Sep;57:29–34. doi: 10.1016/j.dnarep.2017.06.022 (PMC5576915; doi:10.1016/j.dnarep.2017.06.022)
Supplement: Fig. S2 [file mmc2.pdf]

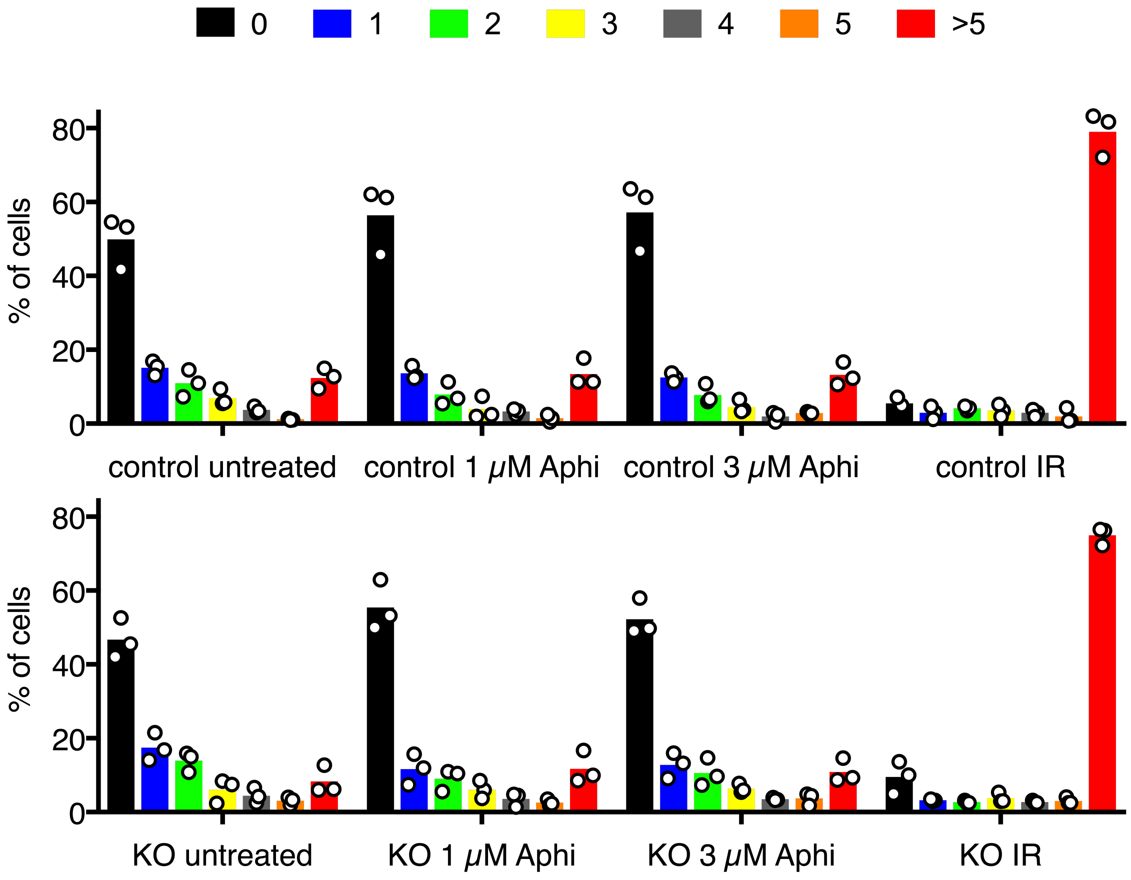

Supplementary Figure S2. Distribution of 53BP1 focus formation. Colours indicated the number of 53BP1 foci per cell according to the colour scheme on the top of the graph. Circles indicate the result for each independent primary MEF preparation.
